# Characterization of Exosomes in Plasma of Patients with Breast, Ovarian, Prostate, Hepatic, Gastric, Colon, and Pancreatic Cancers

**DOI:** 10.4236/jct.2019.105032

**Published:** 2019-05-29

**Authors:** Ming-Bo Huang, Meng Xia, Zhao Gao, Hu Zhou, Min Liu, Shan Huang, Rong Zhen, Jennifer Y. Wu, William W. Roth, Vincent C. Bond, Jian Xiao, Jing Leng

**Affiliations:** 1 Department of Microbiology, Biochemistry and Immunology, Morehouse School of Medicine, Atlanta, Georgia, USA; 2 Guangxi Key Laboratory of Translational Medicine for Treating High-Incidence Infectious Diseases with Integrative Medicine, Guangxi University of Chinese Medicine, Nanning, Guangxi, China; 3 Ruikang Hospital Affiliated to Guangxi University of Chinese Medicine, Nanning, Guangxi, China; 4 Tumor hospital Affiliated to Guangxi Medical University, Nanning, Guangxi, China; 5 The First Affiliated Hospital of Guangxi University of Chinese Medicine, Nanning, Guangxi, China; 6 Columbia College, Columbia University, New York, NY, USA

**Keywords:** Plasma, Mortalin, CD63, Cancer, Extracellular Vesicles, Exosomes

## Abstract

Detection of circulating tumor-specific DNA, RNA or proteins can be difficult due to relative scarcity. Exosomes are extracellular vesicles, 30 – 150 nm in diameter derived from fusion of multivesicular bodies with the plasma membrane. They are composed of a lipid bilayer membrane and contain proteins, mRNA and miRNA. Exosomes are secreted by multiple cell types, including cancer cells. However, there is a relative lack of information concerning the contents of exosomes secreted by various tumor cell types. To examine exosomes in cancer, we collected blood plasma samples from patients with breast, ovarian, prostate, hepatic, gastric, colon, and pancreatic cancers. Exosomes were isolated from plasma and confirmed by AchE assay, transmission electron microscopy and expression of the CD63 exosomal marker. Expression of AFP, CA724, CA153, CEA, CA125, CA199 and PSA antigens were determined using an automated electro-chemiluminescence assay. Expression of the tumor-related chaperone protein, mortalin, was determined by Western blot analysis. Levels of exosome secretion were variable among the different tumor types. Both exosome levels and mortalin expression within tumor cell exosomes were higher than in healthy donors, except in pancreatic carcinoma, where exosomes were elevated but mortalin expression was not significantly different from healthy donors. Exosomes provide unique opportunities for the enrichment of tumor-specific materials and may be useful as biomarkers and possibly as tools of cancer therapies. Mortalin, which has been linked to cell proliferation and induction of epithelial-mesenchymal transition of cancer cells, may be useful as a prognostic bio-marker and as a possible therapeutic target.

## 1. Introduction

Exosomes are cell-secreted extracellular vesicles (EVs) between 30 – 150 nm in size with a closed double-layer membrane structure [1] [2] [3]. Exosomes participate in different biological processes, including mediation of cell-cell signaling by carrying genetic materials between cells [4]. Exosomes exist in virtually all body fluids, including serum [5], normal and malignant urine [6], plasma, breast milk, saliva [7], malignant pleural effusions [8], bronchial lavage fluid [9], ocular samples, tears [10], nasal lavage fluid [11], semen [12], synovial fluid [13], amniotic fluid, and pregnancy-associated serum [14] and carry various molecules (proteins, lipids, and RNAs) on their surfaces as well as in the lumen [1] [2] [3]. Exosomes also contain functionally active proteins, mRNA and miRNA, which can render them important mediators of intercellular communication. Johnstone [15] in 1987 first isolated and purified the exosomes, and the exosomes were found to have the function of removing the redundant cell metabolic components. After nearly 30 years of research, exosomes from different types of cells have been shown to enclose different proteins that have important roles in their biogenesis and are used as makers for their recognition in experimental procedures. Some examples of these proteins are members of the Rab GTPase family [16], tetraspanins (CD9, CD81 [17] and CD63 [18]), and molecular chaperones (heat shock proteins HSP70 [19] and HSP90 [20]). Exosomes have played a critical role in intercellular communication and cellular content transfer, e.g. mRNAs and microRNAs, in both physiological and pathological settings. Their role is verified not only in cellular physiology, but also as playing an important role in tumor progression [21] [22] [23] [24]. The discovery of exosomes represents a research milestone in medicine. In 2013, the achievement regarding exosomes was rewarded with The Nobel Prize, and then more and more people became concerned about the research relative to exosomes.

Release of the extracellular vesicles/exosomes has been reported from a variety of tumor cells [25] [26] [27] [28]. The secreted exosomes can attach to distinct receptors on the surfaces of target cells, releasing their components into the target cells after fusing with their membranes, triggering functional changes within target cells. Zitvogel and colleagues [29] first found that exosomes have functional MHC, and T-cell and costimulatory molecules. The extracellular vesicles/exosomes were found to prime specific cytotoxic T-lymphocytes *in vivo* and to eradicate or suppress growth of established murine tumors in a T cell-dependent manner, suggesting that exosome-based cell-free vaccines could become a therapy against tumors [30]. For the terminal stage of cancer, exosomes derived from tumors have an adverse effect on the immune response. Putz [31] found that the tumor suppressor PTEN is exported in exosomes and has phosphatase activity in recipient cells.

All in all, exosomes contain a wide range of molecules and proteins. Current research [32] [33] [34] suggests the expression patterns of these proteins can be useful in the diagnosis and/or prognosis of cancer. Exosome-contained molecules and proteins could be used as non-invasive biomarkers for the diagnosis, treatment and prognosis in cancer patients since it is easy to obtain the peripheral blood. The characterization and manipulation of exosomes is an important way to establish an exosome-based new clinical approach for diagnosis and treatment of cancers.

Mortalin, also known as GRP75, is a highly conserved molecular chaperone in the heat shock protein (HSP) 70 family, which is encoded by the nuclear gene HSPA9, localized on chromosome [35]. It plays an important role in human carcinogenesis by enhancing cancer cell proliferation, protecting cancer cells against apoptosis and promoting cancer angiogenesis [36]. Overexpression of mortalin may also interact with the wild-type tumor suppressor protein, p53, modulating the Ras-Raf-MAPK pathway and then increasing the malignancy of tumor cells [37] [38]. Mortalin is elevated in human brain tumors, colon carcinoma, leukemia and the immortalized cell lines derived from the tumors [36]. Mortalin also induces cell death and growth arrest in medullary thyroid carcinoma cell lines and mouse xenografts [39]. This study confirms that mortalin is expressed at high levels in several types of cancer cells, except pancreatic carcinoma.

In this study, we measured exosome release into plasma by acetylcholinesterase (AchE) assay, Western blot analysis and electrochemiluminescence analysis. We present data to profile protein expression of exosomes isolated from human blood of cancer and healthy controls, as a proof of principle. The exosomes were purified from plasma of 8 breast cancer patients, 6 ovarian cancer, 14 prostate cancer, 16 hepatocellular carcinoma, 8 gastric carcinoma, 9 colon cancer, and 8 pancreatic carcinoma patients, and isolated exosomes from plasma of these patients and from plasma of healthy donors as a negative control. We measured the CD63 to identify exosomes and used mortalin antibody via Western blot analysis to measure mortalin protein expression level from several types of cancer patient plasma. We also used an automated electrochemiluminescence assay to measure the tumor-specific protein concentration in the exosomes isolated from plasma of patients with the several types of cancer. This research will be helpful for the development of cancer diagnostics and therapeutics.

## 2. Materials and Methods

### 2.1. Reagents and Antibodies

ExoQuick Plasma prep and Exosome precipitation kit were purchased from System Biosciences Inc. (Palo Alto, California, USA), MiRCURY™ Exosome Isolation Kit-Serum and Plasma were purchased from EXIQON (Woburn, MA, USA), SDS-PAGE Sample Loading Buffer 5X, Bradford Protein Assay kit and Nitrocellulose membrane were purchased from Beyotime Inc. (Shanghai, P. R. China). Precision Plus Protein™ Kaleidoscope™ Standards, Precision Protein™ StrepTactin-HRP Conjugated and Precision Protein StrepTactin-AP Conjugate were purchased from BIO-RAD Inc. (Hercules, California, USA), ExpressPlus™ PAGE Gels, Tris-MOPS-SDS Running Buffer Powder and Transfer Buffer Powder were purchased from GenScrit Inc. (Nanjing, Jiangsu, P. R. China), Stripping Buffer was purchased from CWBio Inc. (Shanghai, P. R. China). The CD63 rabbit polyclonal antibody was purchased from Santa Cruz Biotechnology Inc. (Santa Cruz, California, USA). CEA monoclonal antibody, Pierce Goat Anti-Rabbit IgG, (H + L), Peroxidase Conjugated and ECL buffer were purchased from ThermoFisher Scientific Inc. (Rockford, IL, USA), anti-Cytokeratin 5 antibody, anti-HE4 antibody, Anti-MUC1 antibody, anti-alpha 1 Fetoprotein antibody, anti-CA19–9 antibody and anti-Grp75 (mortalin) antibody were purchased from Abcam Inc. (Cambridge, MA, USA).

### 2.2. Patients and Plasma Samples

Sixty-nine patients diagnosed with primary cancer in Ruikang Hospital Affiliated with Guangxi University of Chinese Medicine Hospital and Tumor Hospital Affiliated with Guangxi Medical University were recruited for this study (Table 1). Human peripheral blood samples were obtained from three control healthy subjects and from the sixty-nine cancer patients assigned to seven groups representing different types of tumors: hepatocellular carcinoma (HCC), gastric cancer (GC), breast cancer (BC), colon cancer (CC), ovarian cancer (OC), pancreatic cancer (PC), prostate cancer (PST) with distant metastasis, or from patients without distant metastasis at Guangxi University of Chinese Medicine, the First Affiliate hospital and the affiliate Ruikang Hospital, all pathologically confirmed. All patients were undergoing treatment at the time of study enrollment, except for two of the hepatocellular carcinoma (HCC) patients who were sampled prior to the beginning of treatment (Table 1). This study was performed with the approval of the Ethics Committee of Guangxi University of Chinese Medicine, P. R. China. Patients were exposed to no additional risks or treatments as a consequence of participation in this study. All individuals provided informed consent for blood donation. Briefly, 5 – 8 mL blood was collected in a 10 mL EDTA routine blood tube, spun at 2000 × g for 30 minutes at 4°C with the Eppendorf centrifuge (Eppendorf Centrifuge 5430R, Millipore Corporation, USA), and then the supernatant (plasma) was collected in 2-mL-frozen tube and stored at −80°C.

### 2.3. Isolation and Purification of Exosomes from Human Plasma

The plasma samples from cancer patients and samples from healthy volunteers were separately pooled in order to perform isolation of exosomes by Exosome Isolation Kit (Exiqon, Woburn, MA, USA) or ExoQuick Plasma prep and Exosome precipitation kit (SBI, Palo Alto, CA, USA). For isolation of 1 mL exosomes from plasma using the exosome-isolation reagent, plasma was added to 17 μL of Thrombin, mixed and incubated for 5 minutes at room temperature, and then was centrifuged at 10,000 × g for 5 minutes at 4°C using Eppendorf Centrifuge 5430 R to remove the cell debris. The exosome supernatants were collected and combined with 560 μL of precipitation buffer A, vortexed for 5 seconds to mix and placed at 4°C overnight. After incubation, the mixture was centrifuged at 3200 × g for 30 minutes at 4°C to get the exosome pellet. Finally, after removing the supernatant, the exosome pellet was resuspended in 1 mL of PBS for Western blotting and frozen at −80°C.

### 2.4. Exosome Characterization by Acetylcholinesterase (AchE) Assay

Purified exosomes were quantitated by measurement of AchE as described [40]. Briefly, we prepared 100 mM dithiobisnitrobenzoic acid (DTNB) as a stock color indicator and prepared 28.9 mg/mL in PBS of acetylthiocholine iodide as a stock substrate. Substrate stock can be stored at −20°C up to one month and color indicator can be stored at 4°C for two weeks. A working solution was prepared by mixing 10 mL of PBS with 200 μL of substrate and 500 μL of DTNB. 50 μL of each exosome sample was transferred to 96 well microtiter plates, and then a standard curve was prepared using AchE concentrations from 0.98 mU/mL to 2000 mU/mL. After 50 μL of standards were added into separate wells, we added 200 μL of the working solution to all wells. After 20 min incubation, AchE activity was measured at 450 nm using Gen5 software (BioTek Instruments, Inc, Winooski, VT, USA).

### 2.5. Western Blot Analysis of Exosome Proteins

Exosomes were lysed using lysis buffer. Protein lysates of exosomes (20 μg) were run on 4% – 20% Mini-PROTEIN TGX gel (Bio-Rad, Hercules CA, USA) and transferred to Nitrocellulose membrane. The blots were incubated separately either with rabbit polyclonal anti-human CD63 antibody (Santa Cruz Biotechnology Inc. Santa Cruz CA, USA) at a dilution of 1:500 or rabbit polyclonal anti-human Grp-75 (mortalin) antibody (Cat# ab53098, Abcam, Cambridge MA, USA) at a dilution of 1:1000, and incubated at 4°C for overnight followed by washing with TBS buffer. The blots were incubated with secondary antibody, either horseradish peroxidase (HRP)-conjugated goat anti-mouse (Cat# 31460) from ThermoFisher Scientific (Rockford IL, USA) at a dilution of 1:10,000 (5% dry Milk containing TBS buffer) for 1 hour at room temperature. The blots were washed with TBST at room temperature for 1.5 hours (washing/30 min, 3×), and then were treated with the 1:1 ECL buffer A and B (ThermoFisher Scientific Inc.) according to the user manual, developed on image instrument and finally observed using ChemiDoc MP Imaging System Image Lab software (Bio-Rad, USA).

### 2.6. Evaluation of Tumor Markers by Electrochemiluminescence (ECL) Assay

Twenty micrograms (20 μg) of the exosome and plasma samples were diluted with 300 μL PBS, and the marker protein of each cancer was measured using the Cobas E601 Auto DELFIA automated chemiluminescence system (Roche Diagnostics, Basel, Switzerland). The antibodies against the following proteins were used: AFP (Roche Diagnostics 21371601), CA724 (Roche Diagnostics, 18914001), CA153 (Roche Diagnostics 20990001), CEA (Roche Diagnostics 16842403), CA125 (Roche Diagnostics 18748901), CA199 (Roche Diagnostics 16483403) and PSA (Roche Diagnostics 19942201). Electrochemiluminescence (ECL) is a kind of luminescence produced during electrochemical reactions in solutions. The Cobas E601 Analyzer is a fully automated discrete immunoassay analyzer intended for the in vitro quantitative/qualitative determination of analytes in body fluids. The levels of AFP, CA724, CA153, CEA, CA125, CA199 and PSA levels were measured using the standard protocols recommended by the manufacturer. The recommended antibodies values for diagnostic purposes were: 1) R1 75 μL of 4.5 mg/L and R2 79 μL of 12.0 mg/L for AFP; 2) R1 65 μL of 1.0 mg/L and R2 67 μL of 6.0 mg/L for CA724; 3) R1 74 μL of 1.75 mg/L and R2 73 μL of 1.0 mg/L for CA153; 4) R1 of 80 μL of 3.0 mg/L and R2 61 μL of 4.0 mg/L for CEA; 5) R1 70 μL of 1.0 mg/L and R2 73 μL of 1.0 mg/L for CA125; 6) R1 69 μL of 3.0 mg/L and R2 80 μL of 4.0 mg/L for CA199, and 7) R1 70 μL of 1.5 mg/L and R2 73 μL of 1.0 mg/L for PSA. In the first incubation 20 μg of sample, biotinylated specific antibody, and specific antibody labeled with a ruthenium complex react to form a sandwich complex. In the second incubation, after addition of streptavidin-coated microparticles, the complex becomes bound to the solid phase via interaction of biotin and streptavidin. The reaction mixture is then aspirated into the measuring cell where the microparticles were magnetically captured onto the surface of the electrode. Unbound substances were then removed with ProCell M. Application of a voltage to the electrode then induces chemiluminescent emission which is measured by a photomultiplier. Results are then determined via a calibration curve which is specifically generated by 2-point calibration and uses a master curve provided by automated scanning of the reagent barcode.

### 2.7. Electron Microscopy

For EM studies, 1.67 μg of exosomes (in 10 μL) were added into the front of the copper mesh, left at room temperature for 1 – 2 minutes, and filtered via filter paper to remove excess liquid; 30 μL of 2% phosphotungstic acid was added into the copper wire, incubated at room temperature for 30 seconds, and filtered via filter paper to remove the excess dye solution; it was dried at room temperature for 5 – 10 min; finally, it was observed under a transmission electron microscope (TEM) (Japan, HITACHI, H-7650 type).

### 2.8. Statistical Analysis

Descriptive data were expressed as Means ± Standard Error of Mean (SEM). Independent sample t-test was used. A p-value < 0.05 was considered as significant. Statistical analysis was performed using SPSS statistics software, version 11.5.

## 3. Results

### 3.1. Specific Tumor Cell Markers were Expressed in Exosomes

**Figure 1.**
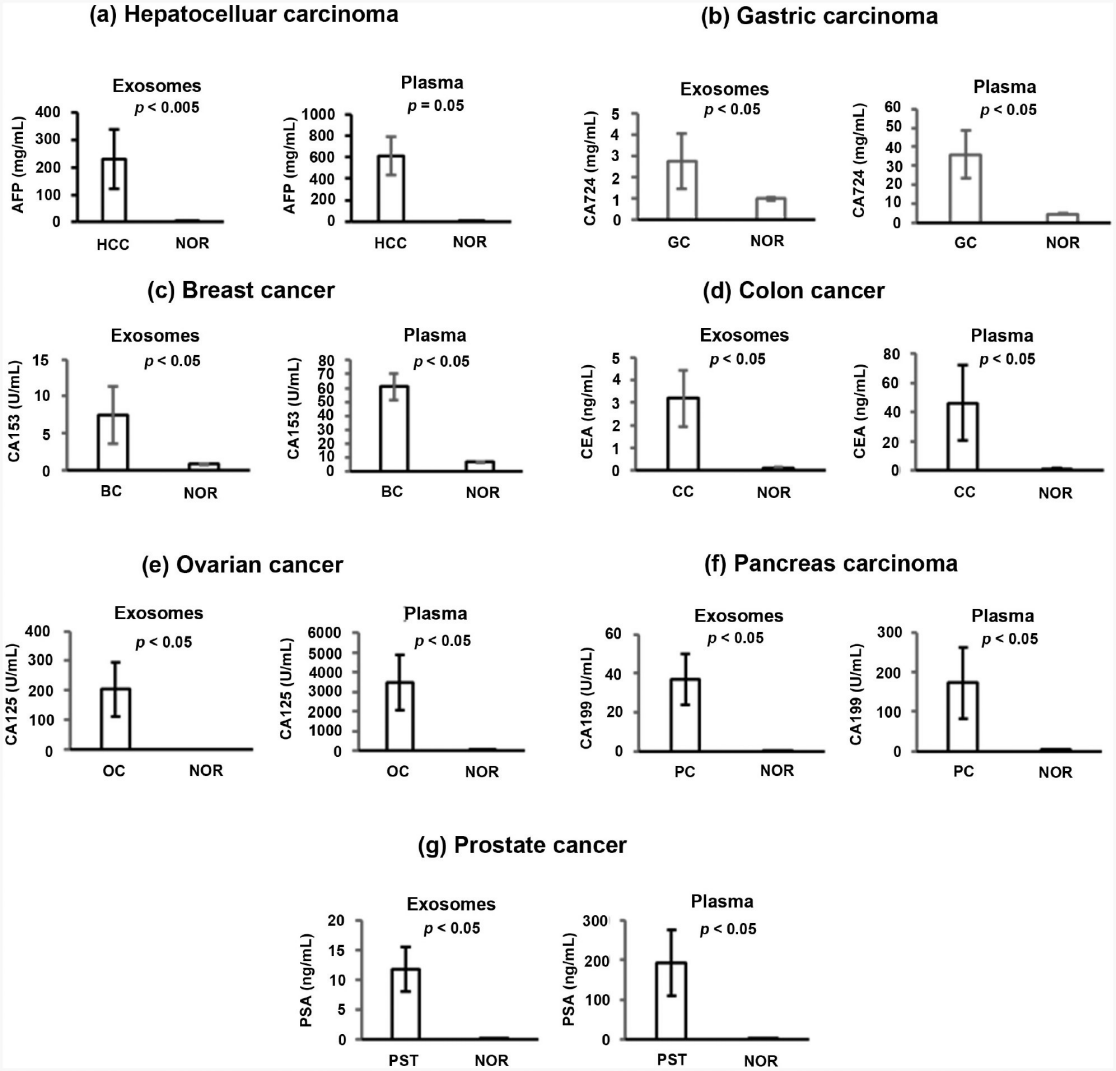
Protein analysis of cancer patients by electrochemiluminescence (ECL) assay. Bar graphs show protein expression levels of the specific tumor-cell markers AFP, CA724, CA153, CEA, CA125, CA199 and PSA in unfractionated plasma and exosomes of their respective groups of patients. Error bars represent the mean ± SEM of three separate assays. A p-value of < 0.05 was considered significant.

An automated microfluidic electrochemiluminescence device was used for accurate, sensitive measurements of specific tumor markers including: Prostate specific antigen (PSA), hepatocellular carcinoma Alpha-fetoprotein (AFP) tumor marker, breast cancer antigen (CA153), colon cancer antigen (CEA), gastric carcinoma (CA724) tumor maker, ovarian cancer (CA125) tumor marker, and pancreas carcinoma (CA199) tumor marker in plasma and exosome. CA125 and CA72–4 are members of a family of high-molecular-weight glycosylated proteins and are commonly considered as biomarkers in the diagnosis of ovarian and gastric cancer, respectively. Recent clinical studies have revealed that these two markers plus CA199 may be of clinical value in the diagnosis of pancreatic cancer. We found that AFP, CA724, CA153, CEA, CA125, CA199 and PSA were expressed in both unfractionated plasma and exosomes of patients with hepatocellular carcinoma, gastric carcinoma, breast cancer, colon cancer, ovarian cancer, pancreatic carcinoma and prostate cancers, respectively. These proteins, as expected, were significantly higher (p < 0.05) compared with plasma and exosomes of healthy donors (Figure 1).

### 3.2. Exosomes were Increased in Plasma of Cancer Patients

**Figure 2.**
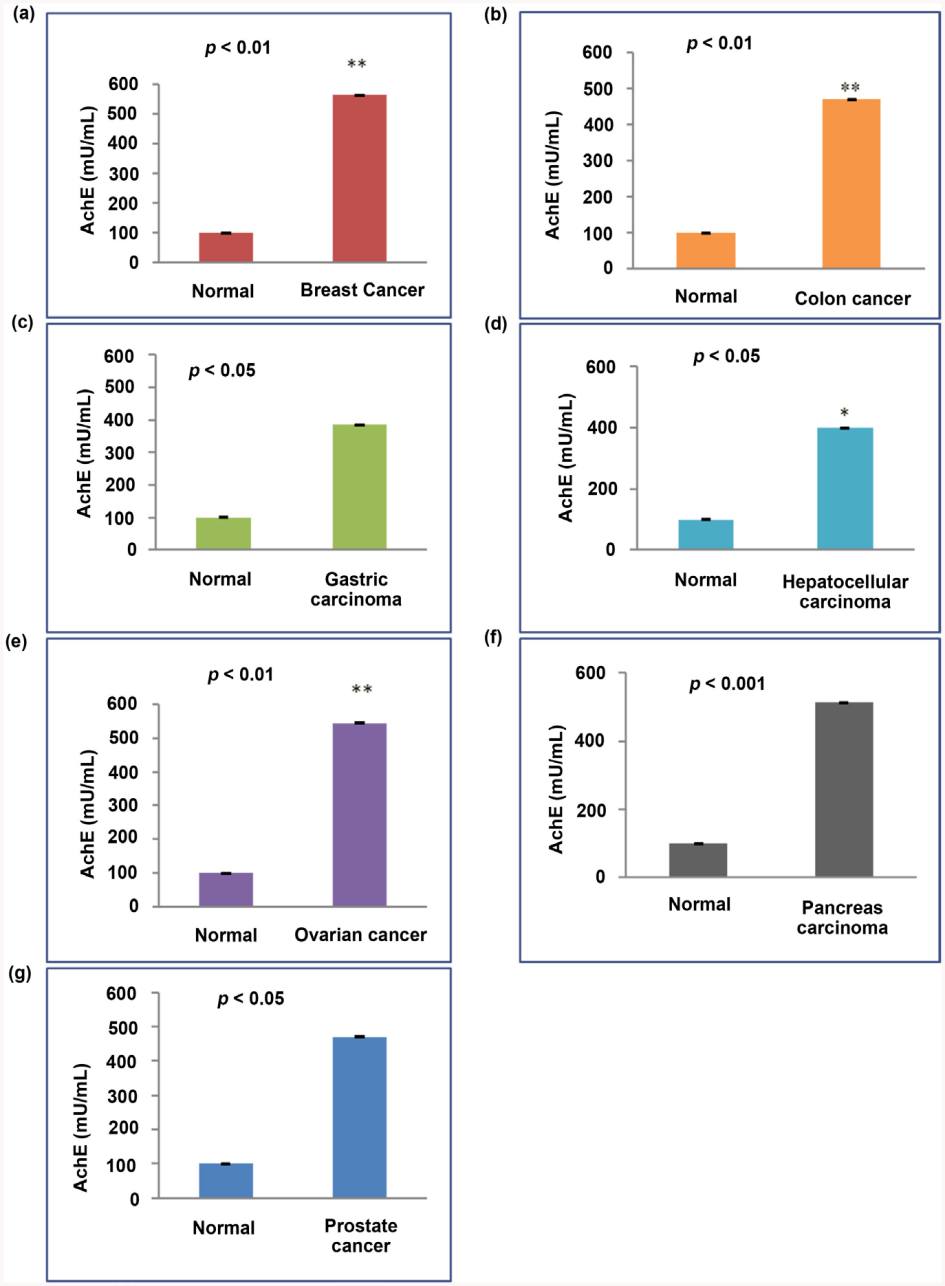
Analysis of exosomes from cancer patients by AchE assay. Bar graphs show relative levels of exosomes in each group of patients compared to normal donors as measured by AchE assay. Error bars represent the mean ± SD of three separate assays. Asterisks (*) indicate significant differences (p < 0.05) relative to each comparison. *p < 0.05, **p < 0.01, ***p < 0.001.

**Figure 3.**
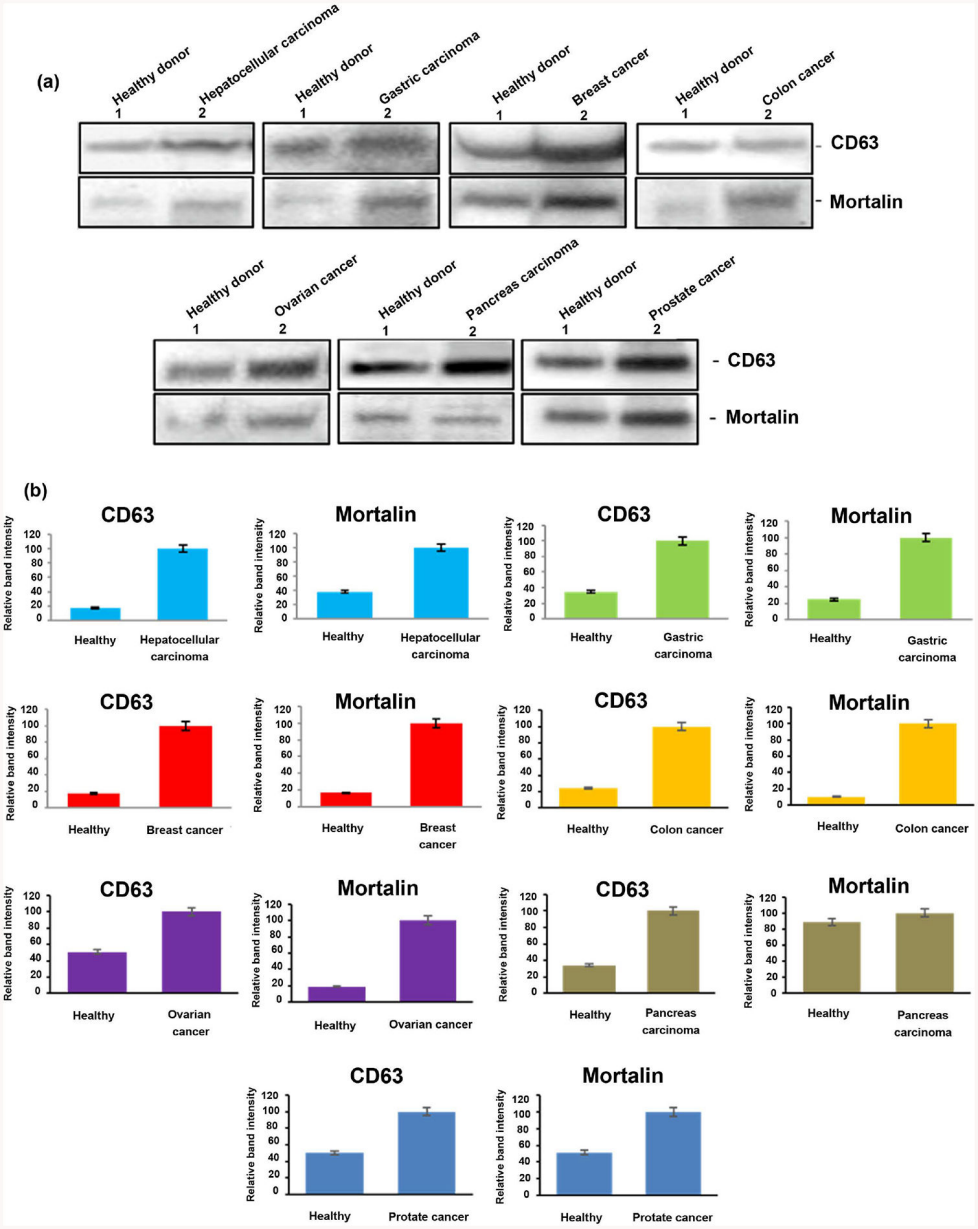
CD63 and mortalin expression in exosomes of cancer patients and healthy donors. Exosome proteins were electrophoresed and blotted as described in the Methods. Gel images (a) and bar graphs (b) from scanned gel images show levels of CD63 and mortalin (GRP75) proteins in exosomes from patients with each of the seven different cancers, compared to healthy donor controls.

We performed acetylcholinesterase (AchE) assays and Western blot analysis in order to compare protein abundance in exosomes purified from the plasma of patients and normal volunteers and measure exosomes released from cancer patients. The results indicated that exosome release was increased in breast cancer, colon cancer, gastric carcinoma, hepatocellular carcinoma, ovarian cancer, pancreas carcinoma and prostate cancer (Figure 2). We also confirmed the identity of exosomes with exosome marker CD63 by Western blot. Western blot analysis demonstrated that all seven types of tumor cell exosomal preparations were enriched in the exosome marker CD63 (Figure 3). We observed a significant difference of CD63 protein levels between two groups of exosome samples derived from healthy people and cancer patients p < 0.01 for hepatocellular carcinoma patients, p < 0.001 for gastric carcinoma patients, p < 0.001 for breast cancer patients, p < 0.0001 for colon cancer patients, p < 0.001 for ovarian cancer patients, p < 0.001 for pancreas carcinoma and p < 0.0001 for prostate cancer patients) (Figure 3).

### 3.3. The Chaperone Protein Mortalin is Expressed in Exosomes of Cancer Patients

To verify whether mortalin expression levels were increased in exosomes from the seven different types of cancers, we performed Western blots to compare exosomes from patients and normal donors. We observed a significant differences in mortalin levels between exosomes derived from healthy people and cancer patients (p < 0.001 for hepatocellular carcinoma patients, p < 0.00001 for gastric carcinoma patients, p < 0.00001 for Breast cancer patients, p < 0.0001 for colon cancer patients, p < 0.0001 for ovarian cancer patients, p < 0.001 for and p < 0.001 for prostate cancer patients). The increase in mortalin expression was especially evident in breast cancer. Interestingly, we did not observe a significant difference in mortalin protein levels between the exosomes from pancreas carcinoma patients and healthy controls p = 0.09 (Figure 3).

### 3.4. Morphological Characteristics of Exosomes

**Figure 4.**
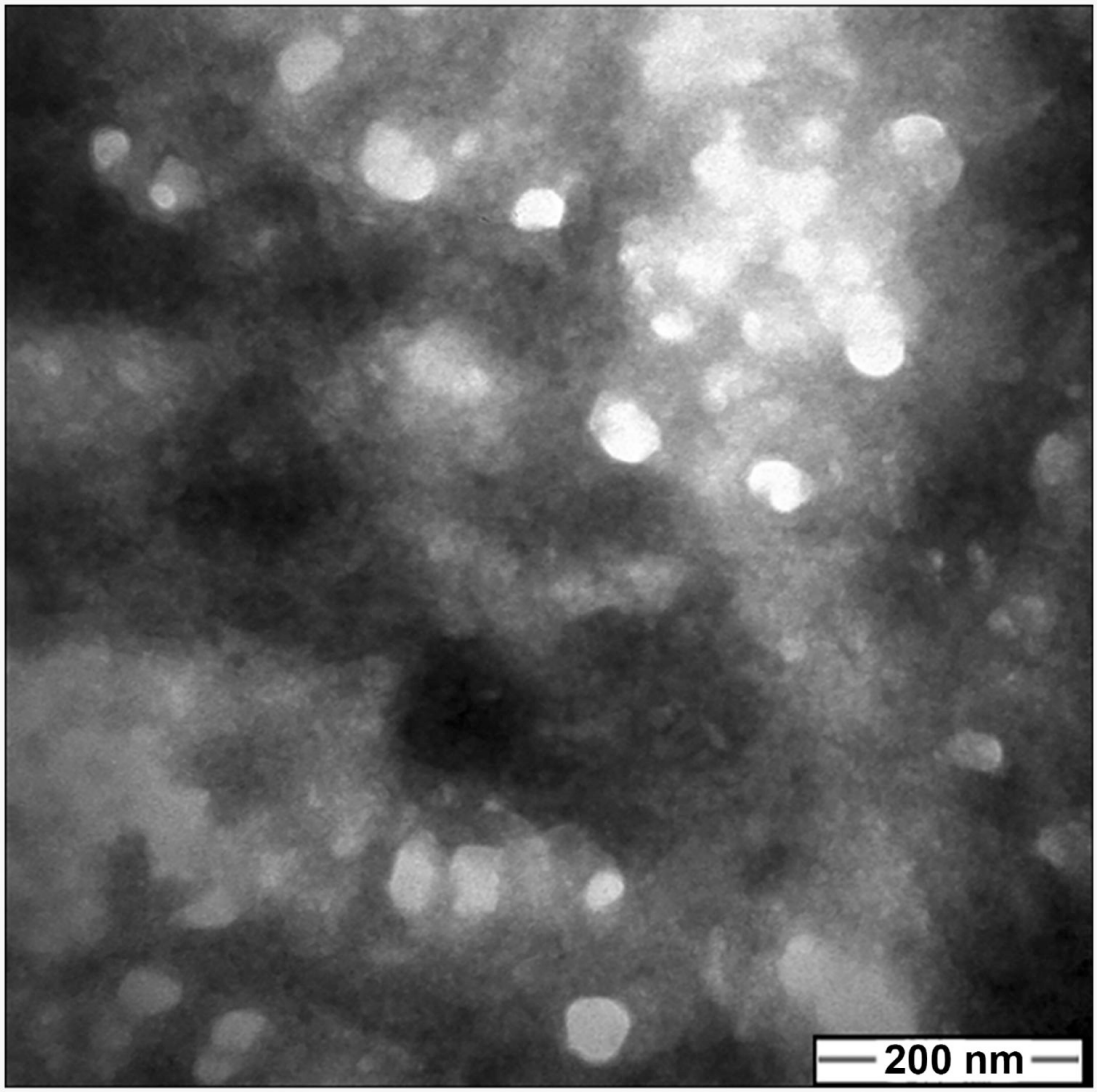
Morphological characteristics of exosomes from human blood plasma. Exosomes were prepared as described in Methods. Observation of the morphology of exosomes from a hepatocellular carcinoma patient by transmission electron microscopy (TEM) indicates the diameter of isolated exosomes is 50 – 100 nm.

Observation of the morphology of exosomes by transmission electron microscopy (TEM) indicated that the diameter of exosomes prepared from tumor cells ranged from 50 – 100 nm (Figure 4), which is consistent with previously reported size range for exosomes.

## 4. Discussions

In this investigation, we focused on the expression in exosomes of specific tumor-associated antigens and mortalin (mthsp70/Grp75), a molecular chaperone member of the heat shock protin (HSP) 70 family, which was shown to be enriched in human cancer cells [36] [41] [42]. It has been established that mortalin has been found in various subcellular localizations, interacts with multiple binding partners and likely plays a role in carcinogenesis. Mortalin has been assigned multiple functions ranging from stress response, intracellular trafficking, antigen processing, as well as control of cell proliferation, differentiation and tumorigenesis.

Increased expression of mortalin is significantly associated with tumor transformation. Mortalin may block apoptosis and help cells to adapt to adverse microenvironments as mentioned above, or may chaperone the mutated proteins of cancer cells, such as mutated p53, which is observed in approximately 50% cancers [45] [46]. Moreover, the level of mortalin is reportedly increased in a variety of tumor cells or tissues, as compared to normal cellular levels [47] [42]. Several studies have shown the importance of the interaction between GRP75 and p53 in carcinogenesis [48] [49]. Therefore, suppression of the gene HSPA9 expression or interference with interaction would be a likely therapeutic strategy against cancer. Indeed, knockdown of the gene HSPA9 by ribozyme or siRNA was shown to reduce growth and viability of human cancer cells and to decrease exosome release [28] [50] [51]. Disrupting the interaction between GRP75 and p53 by the potent anti-cancer drug, MKT-077 [52] or GRP75 binding peptide activates endogenous p53, thus preventing cell growth of osteosarcoma and breast carcinoma cells [49]. Targeting mortalin by siRNA resulted in growth arrest of cancer cells and reduced exosome release by MCF-7 breast cancer cells [28].

In this study, we confirmed that the level of mortalin was elevated in plasma-derived exosomes of patients with different types of tumors, which supports the premise that increased expression of mortalin may promote human carcinogenesis, with the possible exception of pancreatic carcinoma. Compared with healthy donors, using Western blot analysis, the chaperone protein mortalin (GRP75) was found to be significantly upregulated in hepatocellular carcinoma (HCC), gastric cancer (GC), breast cancer (BC), colon cancer (CC), ovarian cancer (OC), and prostate cancer (PSC) exosomes. Recent studies have shown that the expression levels of mortalin in cell lines with higher metastatic potential were significantly higher compared to those with lower metastatic potential. Compared with normal liver tissues, the expression of mortalin was significantly increased in hepatocellular carcinoma tumor tissues [43]. Lu *et al*. showed that human HepG2 cells lacked Mortalin p53 interaction and were resistant to apoptosis, but cell apoptosis was significantly increased by Mortalin shRNA transfection [44]. Chen *et al*. also showed that low expression of mortalin was able to inhibit EMT, decrease tumor progression and lose the metastasis-inducing capability [43]. Despite these interesting findings in our study, a larger sample size in randomized studies is needed to assess further the potential value of mortalin as a candidate biomarker for cancer surveillance.

## 5. Conclusion

Exosomes are drawing increased attention as a potential source to discover new biomarkers for different diseases including cancer. The ideal cancer biomarker can indicate the existence of a tumor in the early stages. Extracellular vesicles such as exosomes have special properties, which make them ideal tools for minimally invasive liquid biopsies. These subcellular particles are detectable in many different biofluids; therefore, in accordance with the type of cancer, we can use these biofluids to detect patients’ extracellular vesicles/exosomes. Our data indicated that exosome levels are increased in patients with different types of tumors including hepatocellular carcinoma (HCC), gastric cancer (GC), breast cancer (BC), colon cancer (CC), ovarian cancer (OC), pancreas carcinomas (PC) and prostate cancer (PST). These exosomes express high levels of the known tumor-specific antigens. Many cancer-related proteins found in tumor-derived exosomes are known for their roles in cancer development and progression. In this regard, we showed that the chaperone protein mortalin is also expressed at high levels in exosomes from patients. Thus, it might also be an attractive biomarker for prognostic evaluation and a potential molecular therapeutic target in patients with several different types of cancers.

## Figures and Tables

**Table 1.** Cancer patient blood sample sources.

Cancer	Quantity	Age-Bracket	Hospital Sources
Hepatocellular Carcinoma	14	35 – 77	10 from Ruikang Hospital Affiliated with GUCM* & 4 from the First Affiliated Hospital with GUCM
Hepatocellular Carcinoma (Untreated)	2	41 – 47	1 from Ruikang Hospital Affiliated with GUCM & 1 from the First Affiliated Hospital with GUCM
Ovarian Cancer	6	31 – 74	1 from Ruikang Hospital Affiliated with GUCM & 5 from the First Affiliated Hospital with GUCM
Colon Cancer	9	38 – 68	3 from Ruikang Hospital Affiliated with GUCM & 6 from the First Affiliated Hospital with GUCM
Gastric Cancer	8	55 – 82	4 from Ruikang Hospital Affiliated with GUCM & the First Affiliated Hospital with GUCM
Breast Cancer	8	36 – 71	4 from Ruikang Hospital Affiliated with GUCM & 4 from the First Affiliated Hospital with GUCM
Prostate Cancer	14	59 – 85	4 from Ruikang Hospital Affiliated with GUCM & 5 from the First Affiliated Hospital of GUCM & 4 from the Peoples Hospital of Guangxi Zhuang Autonomous Region & 1 from the First Affiliated Hospital with Guangxi Medical University
Pancreas Carcinoma	8	35 – 73	2 from Ruikang Hospital Affiliated with GUCM & 2 from Tumor Hospital Affiliated with Guangxi Medical University & 2 from the Peoples Hospital of Guangxi Zhuang Autonomous Region & 1 from the First Affiliated with Guangxi Medical University
Normal Subjects	3	23 – 27	Healthy Donors

* Guangxi university of chinese medicine.
